# Enrichment of Circular RNA Expression Deregulation at the Transition to Recurrent Spontaneous Seizures in Experimental Temporal Lobe Epilepsy

**DOI:** 10.3389/fgene.2021.627907

**Published:** 2021-01-28

**Authors:** Andreia Gomes-Duarte, Sebastian Bauer, Morten T. Venø, Braxton A. Norwood, David C. Henshall, Jørgen Kjems, Felix Rosenow, Vamshidhar R. Vangoor, R. Jeroen Pasterkamp

**Affiliations:** ^1^Affiliated Partner of the European Reference Network EpiCARE, Department of Translational Neuroscience, University Medical Center Utrecht Brain Center, University Medical Center Utrecht, Utrecht University, Utrecht, Netherlands; ^2^Epilepsy Center Frankfurt Rhine-Main, Neurocenter, University Hospital Frankfurt and Center for Personalized Translational Epilepsy Research, Goethe-University Frankfurt, Frankfurt, Germany; ^3^Epilepsy Center, Department of Neurology, Philipps University Marburg, Marburg, Germany; ^4^Interdisciplinary Nanoscience Centre, Department of Molecular Biology and Genetics, Aarhus University, Aarhus, Denmark; ^5^Omiics ApS, Aarhus, Denmark; ^6^Department of Neuroscience, Expesicor Inc., Kalispell, MT, United States; ^7^Diagnostics Development, FYR Diagnostics, Missoula, MT, United States; ^8^Department of Physiology and Medical Physics, Royal College of Surgeons in Ireland, Dublin, Ireland; ^9^FutureNeuro, The Science Foundation Ireland Research Centre for Chronic and Rare Neurological Diseases, Royal College of Surgeons in Ireland, Dublin, Ireland

**Keywords:** temporal lobe epilepsy, epileptogenesis, circular RNA, microRNA, network analysis, RNA-sequencing, early epilepsy

## Abstract

Mesial temporal lobe epilepsy (mTLE) is a common form of epilepsy and is characterized by recurrent spontaneous seizures originating from the temporal lobe. The majority of mTLE patients develop pharmacoresistance to available anti-epileptic drugs (AEDs) while exhibiting severe pathological changes that can include hippocampal atrophy, neuronal death, gliosis and chronic seizures. The molecular mechanisms leading to mTLE remain incompletely understood, but are known to include defects in post-transcriptional gene expression regulation, including in non-coding RNAs (ncRNAs). Circular RNAs (circRNAs) are a class of recently rediscovered ncRNAs with high levels of expression in the brain and proposed roles in diverse neuronal processes. To explore a potential role for circRNAs in epilepsy, RNA-sequencing (RNA-seq) was performed on hippocampal tissue from a rat perforant pathway stimulation (PPS) model of TLE at different post-stimulation time points. This analysis revealed 218 differentially expressed (DE) circRNAs. Remarkably, the majority of these circRNAs were changed at the time of the occurrence of the first spontaneous seizure (DOFS). The expression pattern of two circRNAs, circ_Arhgap4 and circ_Nav3, was further validated and linked to *miR-6328* and *miR-10b-3p* target regulation, respectively. This is the first study to examine the regulation of circRNAs during the development of epilepsy. It reveals an intriguing link between circRNA deregulation and the transition of brain networks into the state of spontaneous seizure activity. Together, our results provide a molecular framework for further understanding the role and mechanism-of-action of circRNAs in TLE.

## Introduction

Epilepsy is considered the most common chronic brain disease and is estimated to affect more than 50 million people worldwide (Ngugi et al., 2010). Focal epilepsies account for 60% of all epilepsy forms (Devinsky et al., 2018). mTLE is a frequent form of focal epilepsy and is characterized by pathological manifestations that include hippocampal atrophy, neuronal death, gliosis and spontaneous seizures. Several factors have been identified as triggers that may lead to epilepsy, e.g., infectious agents, head trauma, brain tumors and genetic contributions (Duncan et al., 2006). While in the majority of patient symptoms can be controlled by AEDs, some patients do not become seizure-free and/or experience major adverse events in response to treatment (Wieser, 2004; Boon et al., 2009). In mTLE, the proportion of patients who do not become seizure free with AEDs is around 70–90% (Benbadis and Semah, 1999; Kurita et al., 2016). This condition, known as “refractory epilepsy,” highlights the need for the discovery of new molecular pathways that can be therapeutically targeted in TLE.

The molecular mechanisms underlying TLE remain incompletely understood (Dalby and Mody, 2001; Bender et al., 2004; Scimemi et al., 2006). Over the past years, a plethora of studies has shown that ncRNAs can influence various processes in the nervous system, including neuronal development, apoptosis, neurogenesis, oxidative stress, synaptic plasticity, and immune system activation, that are disturbed in the pathological process leading to mTLE (Henshall and Kobow, 2015; Henshall et al., 2016; Shao and Chen, 2017; Villa et al., 2019; Mills et al., 2020). In addition, experimental evidence highlights defects in post-transcriptional regulation in mTLE and the expression and function of different classes of ncRNAs is perturbed in experimental TLE and in TLE patients (Van Gassen et al., 2008; Jimenez-Mateos et al., 2012; Kan et al., 2012; Gong et al., 2018; Lee et al., 2018; Li J. et al., 2018; Guelfi et al., 2019; Fu et al., 2020; Gray et al., 2020; Venø et al., 2020). Together, these observations support the idea that ncRNAs may participate in the process of epileptogenesis. circRNAs are a class of ncRNA molecules that originate from back splicing events of a 5′ splice spite to an upstream 3′ splice site leading to the formation of closed circular structures. The back splicing may occur directly in the primary transcript or in the event of ‘exon skipping,’ where back splicing occurs within the lariat product alongside mRNA production. Further, long introns and inverted repeats in flanking introns are known to potentiate circularization (Jeck and Sharpless, 2014). Due to their circular structure, which lacks a poly-A tail and 5′-cap structure, circRNAs are more resistant to degradation by most RNA decay mechanisms, leading to their accumulation over time (Cortés-López and Miura, 2016; Li X. et al., 2018). Although generally lowly expressed, studies have shown that circRNAs are highly enriched in the normal and diseased brain (Hansen et al., 2011; Rybak-Wolf et al., 2014; Hanan et al., 2017; Li X. et al., 2018). Further, even lowly abundant circRNAs can exert important physiological functions. For example, circ_Spidr can promote axon regeneration upon peripheral nerve injury (Mao et al., 2019).

The mechanism-of-action of most circRNAs remains largely unexplored. Translation into polypeptides and selective interaction with miRNAs and RBPs are some of the currently reported functions of circRNAs (Li X. et al., 2018). Of these proposed functions, the ability of circRNAs to bind miRNAs via partial or full complementarity has been best-characterized. MiRNAs can be involved in post-transcriptional regulation of multiple genes, generally by targeting their 3′ UTRs. Ultimately, this interaction may lead to the activation of the RISC to promote directed miRNA target degradation (Bartel, 2009). Several studies propose a role for circRNAs in regulating miRNA-associated processes through sponging mechanisms, i.e., binding of miRNAs to circRNAs that will prevent these molecules from binding their messenger RNA targets (mRNA) (Hansen et al., 2013; Memczak et al., 2013; Thomas and Sætrom, 2014). It has therefore been proposed that circRNAs may control neuronal mechanisms by indirectly regulating, through miRNA sponging, the degradation or translation mRNA targets (Rong et al., 2017; Kristensen et al., 2019; Guria et al., 2020).

How circRNAs are regulated during the process of epileptogenesis remains unknown. To our knowledge, here we investigate for the first time how a specific circRNA/miRNA/mRNA network is deregulated at early stages of experimental epilepsy using a PPS rat model of TLE.

## Materials and Methods

### Animal Experiments

All animal experiments were approved by local authorities in Marburg (Philips University Marburg, Germany: Regierungspraesidium Giessen, 73/2913) or in Utrecht (Animal Ethics Committee of Utrecht University) in compliance with Dutch laws (Wet op de Dierproeven, 1996; revised 2014). All procedures were performed in accordance with EU regulations (Guideline 86/609/EEC; Directive 2010/63/EU).

Male Sprague-Dawley rats (325–350 g; Charles River) were used in this study. Epilepsy was induced in rats using PPS, as previously described (Norwood et al., 2010; Costard et al., 2019). In short, an EEG transmitter (A3028R-FB, Open Source Instruments, Inc., Watertown, MA, United States) was implanted at the left abdominal site of the rat. Stimulation electrodes (diameter 0.125 mm, Plastics One, Roanoke, VA, United States) were implanted bilaterally into the angular bundle of PP, and recording electrodes (diameter 0.25 mm, Plastics One, Roanoke, VA, United States) were implanted bilaterally into the hilus of the DG. Electrode positions were verified by recording potentials evoked in the DG after PPS.

Starting immediately after surgery, video and EEG were recorded continuously (24/7) for up to 97 days. EEG recordings were performed using an Octal Data Receiver (A3027, Open Source Instruments, Inc., Watertown, MA, United States) with a sampling rate of 512/s. Data were recorded in NDF (Neuroscience Data Format) and converted to EDF (European Data Format) for analysis with EDFBrowser (version 1.57+). Video recording was performed with infrared cameras (IC-7110W, Edimax Technology, Willich, Germany) and sampled with SecuritySpy software (Ben Software Ltd., London, United Kingdom). The total EEG of all rats was screened visually for appearance of seizure patterns. Video was used to clarify appearance of artifacts (e.g., chewing, scratching).

After surgery, a 7-day recovery phase was allowed before bilateral PPS was applied on two consecutive days for 30 min and on the following day for 8 h. The 30 min stimulations on days 1 and 2 induce a state of epileptic tolerance which allows application of 8 h PPS on day 3 without causing self-sustaining status epilepticus or mortality. PPS was performed with a stimulus generator (Grass S88, Grass Telefactor/Grass Instruments, United States) and a stimulus isolator (Model SIU5, Grass Instruments, United States) using the following parameters: pulse duration 0.1 ms, frequency 2 Hz, voltage 20 V, twin pulses with interpulse interval of 40 ms, additional trains of 20 Hz single pulses at 20 V applied once per minute for 10 s. The animals develop mTLE with spontaneous seizure appearing after an average of 19 ± 11 days (Costard et al., 2019). In addition, the animals develop HS ILAE type 1, which corresponds to the most frequently seen HS pattern in human mTLE (Norwood et al., 2010).

Rats were divided into six groups (*n* = 3 rats per group) and were killed by transcardial perfusion with ice-cold PBS at different time points (days): 1 day, 3 days, and 10 days after epilepsy induction by PPS (epileptogenesis); on the day of the first spontaneous seizure (DOFS) (early epilepsy); 30 days after the first spontaneous seizure (chronic epilepsy); and 17 days after surgery (control group), as previously described (Venø et al., 2020). Hippocampi were stored at −80°C until further use.

Wild-type Long Evans rats (7–10 g, Janvier Labs) were used to collect control material for RNase R experiments. Postnatal day 1 (P1) wild-type Long Evans rats were euthanized with Euthanimal 20% (50 mg/kg, i.p., Alfasan) and decapitated. Brains were quickly removed and hippocampi dissected and stored at −80°C.

### Hippocampi RNA Extraction

Frozen hippocampal material was thawed on ice. RNA was obtained using Trizol purification as previously described (PPS model rats) (Venø et al., 2020) or QIAzol lysis reagent with the miRNeasy Mini Kit (Qiagen) according to the manufacturer’s instructions (wild-type Long Evans rats). Purified RNA was stored at −80°C until library preparation (PPS model rats) or measured on Nanodrop (Thermo Fisher Scientific) and stored at −80°C until RNase R experiments were performed (wild-type Long Evan rats).

### Library Preparation, RNA-Sequencing and circRNA Analysis

Purified RNA was rRNA depleted using the Ribo-Zero Magnetic Kit (human/mouse/rat; Illumina). Sequencing libraries were generated using the ScriptSeq v2 kit and sequenced as paired end 100 bp reads on an Illumina HiSeq 4000 sequencer and quality checked using 2100 Bioanalyzer analysis (Agilent). Sequencing data were preprocessed by removing adapter sequences and trimming away low quality bases (Phred score 20) with Trim Galore which uses the Cutadapt algorithm (Martin, 2011). CircRNA detection was performed by mapping filtered reads to the rat genome (rn6) with Bowtie (Langmead et al., 2009), using find_circ (Memczak et al., 2013) to detect BSJ spanning reads from the reads that do not map linearly to the rat genome. Only circRNAs with two or more supporting BSJ reads within single samples were kept. A second circRNA detection algorithm, CIRCexplorer (Zhang et al., 2014), was used to verify the detected circRNAs. CIRCexplorer was guided by Ensembl Release 87 gene annotations on rat genome (rn6). Differential expression analysis was done using DESeq2 (Love et al., 2014) in R, limiting the analysis to the 1000 most highly expressed circRNAs, since these are the most likely to be biologically relevant. Raw counts of BSJ read numbers detected by find_circ were used as input for DESeq2. Statistical metrics produced by DESeq2 including probability value (*p*-value, *p*) and Benjamini–Hochberg adjusted *p*-values are reported with the normalized BSJ expression values generated by DESeq2. Detected circRNAs were checked for conservation in mouse and human by searching for expression of annotated mouse and human circRNAs with identical BSJ after conversion of mouse or human circRNA genomic regions (mm9 or hg19) from circBase^1^ (Glažar et al., 2014) to rat genome (rn6) coordinates using the UCSC liftOver tool (Hinrichs et al., 2006). Homology% analysis was performed by considering the sequence of conserved human circRNAs (as defined by circBase) and using BLAT from UCSC genome browser to match human circRNA sequences to rat genome (Kent, 2002; Glažar et al., 2014). The homology is defined as [matching bp]/[total bp in circRNA]. The complete list of circRNAs detected after RNA-seq and DESeq2 analysis and their specifics can be found in Supplementary Table 1.

### RNase R Treatment, cDNA Synthesis, PCR and Real-Time PCR

Treatment with RNase R was performed for circRNA detection and validation of circularization. Briefly, 5 μg of RNA derived from P1 rat hippocampus was diluted in 20 μl of water containing 4 units/μg (U/μg) of RNase R (Epicenter, Madison, WI, United States), 2 μl of enzyme buffer (Epicenter) and 0,5 μl of RiboLock RNase inhibitor (Thermo Fisher Scientific) followed by incubation for 20 min at 37°C. Retrotranscription of RNA material was performed with SuperScript IV First-strand Synthesis System (SSIV) (Thermo Fisher Scientific) as follows: 500 ng of RNase R treated or untreated RNA was retrotranscribed in a 20 μl reaction mix according to the manufacturer’s protocol and incubated for 10 min at 23°C, 10 min at 53°C and 10 min at 80°C. Semi-quantitative PCR for circularization confirmation following RNase R treatment was performed by amplifying 100 ng of cDNA with 0.3 μl of TAQ^TM^ DNA Polymerase (Qiagen), 2 μl of 10x Reaction Buffer, 4 μl of Buffer Q and 1.6 μl of 10 μM primers in a final 20 μL reaction. Reactions were carried out according to the following program: 95°C for 2 min; 34 cycles at 95°C for 30 s, 60°C for 30 s, 72°C for 1 min and a final extension of 72°C for 10 min. 12 μl of each PCR reaction were run on a 2% agarose gel (m/v) and analyzed using ImageJ software (Schneider et al., 2012). The intensity of each band was calculated as a numerical value meaning the brighter the band the higher the number. Resistance to RNase R treatment was calculated for each transcript as a ratio of the numerical values between the two groups (untreated and RNase R treated). Proof of circularity was defined for each circRNA if the fraction of the RNA expression recovered upon RNase R treatment was superior to the one recovered for the linear reference transcript, *beta-actin* (β*-actin*), with the same treatment.

cDNA used in quantitative real-time PCR (RT-qPCR) for intergroup quantification was processed with SSIV as mentioned above with an additional step. In this case, a spike-in RNA (an RNA transcript of known sequence derived from *Caenorhabditis elegans*) was added to each reverse transcriptase reaction for use in normalization. This allowed detection of possible cDNA synthesis artifacts which could interfere with fold-change (FC) calculations. 2 ng of each cDNA reaction were then used for amplification by RT-qPCR using FastStart Universal SYBR Green Master (Rox) (Sigma-Aldrich) in a QuantStudio(^TM^) 6 Flex System (Thermo Fisher Scientific). The RT-qPCR cycling conditions were as follows: 40 cycles of 95°C for 15 s, 60°C for 1 min and 95°C for 15 s followed by melting curve analysis. All cycle threshold (Ct) values ≥ 35 were considered background or amplification artifacts and discarded from further analysis. Relative expression levels of the analyzed genes were calculated using the 2^–ΔΔCt^ method. For circRNA BSJ sequencing, RT-qPCR reactions were run in a 2% agarose gel (m/v), purified with PureLink^TM^ Quick Gel Extraction Kit (Invitrogen) and further used for custom DNA sequencing (Standard-Seq) with circ_Arhgap4 and circ_Nav3 forward primers (F) (Macrogen).

We designed two different types of primer sets to be used in this study: convergent primers that bind linear mRNA transcripts and divergent primers, intended to bind circRNA molecules that are formed by 3′–5′ back splicing events. All primers used in this study were designed either using the Primer-BLAST online tool^2^ or selected from published literature (*Spidr, Cd200*) (Fonken et al., 2018; Mao et al., 2019) and purchased from IDT-DNA (Supplementary Table 2). Two pairs of primers, β*-actin* and *Gapdh*, were used as housekeeping genes. NormFinder (^∗^.xla, MS Excel 2003 v0.953) was used to determine if a combination of housekeeping genes would be beneficial considering the experimental groups analyzed (Andersen et al., 2004) (Supplementary Table 3). Actin-1 precursor (act-1) primers were used for spike in detection.

### circRNA/miRNA/mRNA Network Construction

circRNA/miRNA/mRNA interaction networks were predicted and built according to a two-method sequential analysis. Initially, the freely accessible miRDB database (Chen and Wang, 2020) was used to predict both miRNA targeting sites in the circRNA sequences and subsequent mRNA targets of selected miRNAs. Targets retrieved from miRDB were predicted by a bioinformatics tool, miRTarget, that combines information from the analysis of multiple miRNA-target interactions from high-throughput sequencing experiments^3^ (Liu and Wang, 2019). In our analysis, the custom prediction function of miRDB^4^ (Chen and Wang, 2020) was used to predict annotated miRNAs with potential binding sites on circ_Arhgap4 and circ_Nav3 sequences in *Rattus norvegicus*. A target score of 60 was used as a threshold for the selection of the predicted miRNAs. Further, the previously selected miRNAs were used in the prediction of potential mRNA targets in *Rattus norvegicus*. Here, a target score of 80 was used as a threshold for target selection, as a prediction score greater than 80 is most likely to constitute a real interaction. The second step of the analysis included the construction of a network composed by the selected miRNA targets predicted during the first step of our pipeline. Cytoscape v_3.7.2 was used to obtain a graphic representation of the circRNA/miRNA/mRNA molecular network (Shannon et al., 2003). All miRNAs (predicted target score > 60) were assigned as source nodes whereas all mRNAs (predicted target score ≥ 80) were assigned as target notes during the network construction.

### Pathway Analysis of circRNA/miRNA/mRNA Genes

g:Profiler^5^ (version e99_eg46_p14_f929183) was used to perform functional profiling of the miRNA targets retrieved previously (Raudvere et al., 2019). g:Profiler output was then used for generating an Cytoscape EnrichmentMap representation of the main biological processes associated with the mRNA network, according to a published pipeline (Reimand et al., 2019).

The enrichment analysis of target genes in biologically relevant pathways was performed with Cytoscape StringApp (Shannon et al., 2003; Doncheva et al., 2019). StringApp uses information from open-source databases to retrieve networks and functional enrichment analysis by combining several selective categories (GO, InterPro Domains, KEGG Pathways, Reactome Pathways, PFAM Domains, etc.). In this study, the STRING: protein query public database was used as data source (Doncheva et al., 2019). The enrichment score associated with each category was calculated as the negative logarithm of the FDR value. The most significant sorted FDR values were used as criteria for the statistical significance of the enrichment found. Relevant genes of interest and corresponding most significant enrichment processes were used for the visual representation of the pathway analysis using Cytoscape StringApp, as described previously.

### Statistical Analysis

GraphPad Prism 8 was used to assess statistical significance for RT-qPCR analysis, which was performed using an unpaired two-tailed Student’s *t*-test; *n* = 3 animals per group. Statistical significance was considered for *p* < 0.05 (^****^p < 0.0001; ^∗∗∗^*p* < 0.001; ^∗∗^*p* < 0.01; ^∗^*p* < 0.05; not significant, ns: *p* ≥ 0.05). All values were expressed as mean ± standard error of the mean (SEM) with the exception of RNase R experiments, in which standard deviation (SD) was used to better represent the variation between independent experiments.

Cytoscape StringApp was used to perform and graphically represent GO analysis results. The FDR method was used in all analyses as a correction for multiple testing according to the Benjamini-Hochberg procedure. Statistical significance was considered for FDR < 0.05.

## Results

### Spatiotemporal circRNA Expression in a Rat PPS Model of mTLE

**FIGURE 1:**
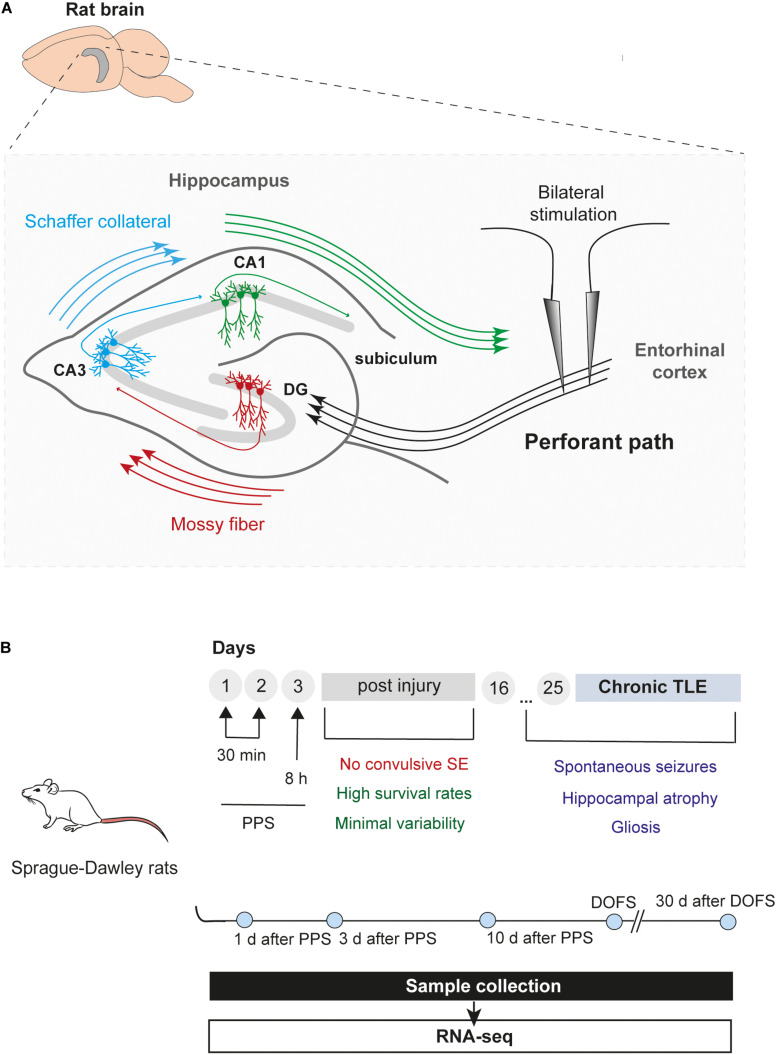
Epilepsy model and timeline of experiments. **(A)** Schematic illustration of the rat hippocampus showing bilateral stimulation of the perforant path. The perforant path is the major axonal connection between the entorhinal cortex (EC) and all hippocampal areas, including the DG (dentate gyrus), CA (*Cornu Ammonis*) regions and subiculum (Witter et al., 2000). Briefly, axons from entorhinal cortex (layer II) project toward the DG and CA3 regions, creating a pathway that propagates through the hippocampus back to the EC (EC – DG – CA3 – CA1 – EC). In addition, granule cells from the DG (“mossy fibers”) can project their axons into the CA3 which, in turn, provides input into the CA1 field via a subset of CA3 fibers (“Schaffer collateral”). Finally, CA1 fibers innervate the subiculum, the final target of the pathway, which incorporates all the input information into the output pathway of the hippocampus (Frotscher et al., 1994) (CA1, *Cornu Ammonis* area 1; CA3, *Cornu Ammonis* area 3; DG, dentate gyrus). **(B)** Overview and timeline of the study, including PPS and sample collection. Rats were divided into six groups: 1 day, 3 days, and 10 days after epilepsy induction by PPS (epileptogenesis); on the day of the first spontaneous seizure (DOFS) (early epilepsy); 30 days after the first spontaneous seizure (chronic epilepsy); 17 days after surgery (control group) (TLE, temporal lobe epilepsy; PPS, perforant pathway stimulation; SE, *status epilepticus*; CA1, *Cornu Ammonis* area 1; CA3, *Cornu Ammonis* area 3; DOFS, day of first spontaneous seizure).

The rat PPS model was used to profile circRNA expression at different stages during the process of epileptogenesis leading to spontaneous seizures and epilepsy (Figure 1A). In this model, seizure activity is provoked and maintained throughout the stimulation process without promoting convulsive SE (Norwood et al., 2011). *In vivo* PPS is characterized by high survival rates and low variability of hippocampal injury while displaying major pathophysiological hallmarks of mTLE, such as hippocampal atrophy, gliosis, hippocampal region-specific neuronal death resembling ILAE type 1, and recurrent spon taneous seizures (Norwood et al., 2010).

**FIGURE 2:**
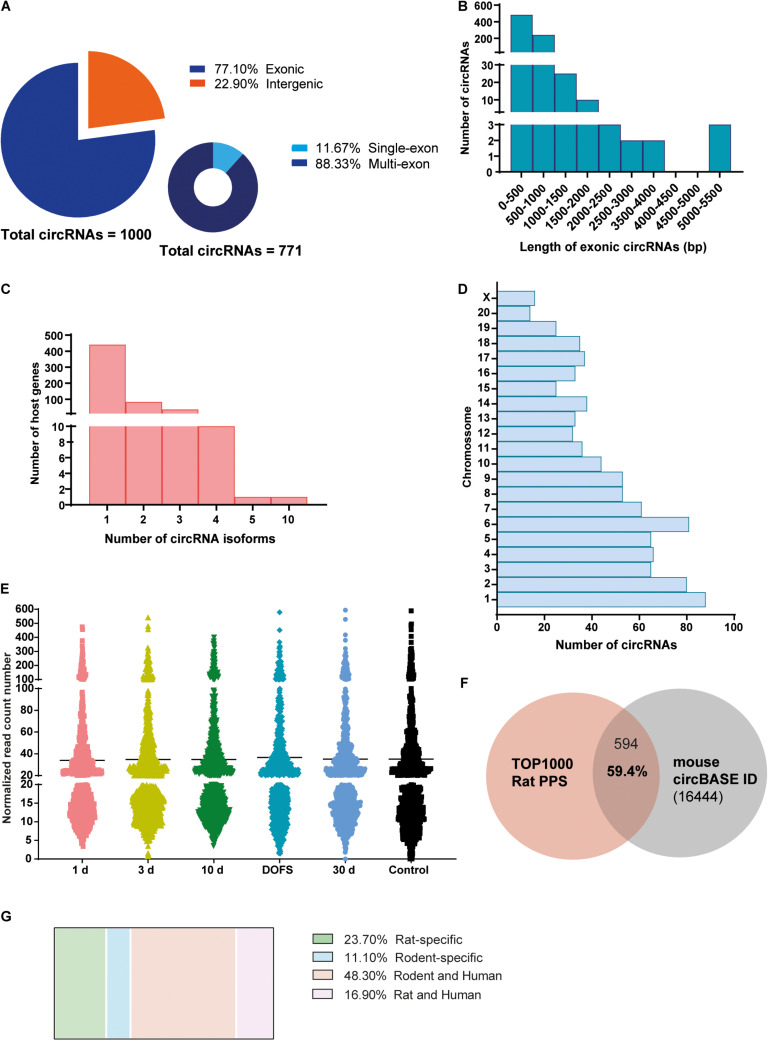
Characterization of circRNA profiling in the PPS rat model. **(A)** Distribution of detected circRNAs in the rat perforant pathway stimulation (PPS) model in different circRNA categories (exonic, intergenic, single-exon and multi-exon). **(B)** Size distribution of different exonic circRNAs, *n* = 771. **(C)** Distribution of the number of circRNA isoforms per host gene. **(D)** Distribution of circRNAs across the different rat chromosomes. **(E)** Expression levels of detected circRNAs across the different experimental groups (1 day, 3 days, and 10 days after PPS, day of first spontaneous seizure (DOFS), 30 days after DOFS and control). Expression levels are shown as the normalized number of RNA-seq reads and each dot represents a specific circRNA. Values are plotted as a scatter to illustrate the spread of data values around the mean (black line). **(F)** Overlap between the 1000 most highly expressed circRNAs detected by RNA-seq and circBase annotated circRNAs (total of 16444 unique *Mus musculus circRNAs*). 59.4% of the circRNAs are conserved in *Mus musculus* and can be found in circBase. **(G)** Species conservation of all detected circRNAs, expressed as% of the total number of circRNAs found.

High-throughput sequencing of rat hippocampi was performed at the following time points: 1 day, 3 days, and 10 days after PPS (epileptogenesis), at the day of the first spontaneous seizure (DOFS) after PPS (early epilepsy), and 30 days after DOFS (chronic epilepsy); 17 days after surgery (control group) (Figure 1B). In total, 22811 circRNAs were identified in both the control and PPS groups. Considering the very low level of expression of several detected circRNAs only the 1000 most highly expressed circRNAs were used in further analyses. Of these, 771 were classified as exonic and 229 classified as intergenic (Figure 2A). Among the exonic circRNAs, the majority was composed of more than one exon (88%), while only 90 (12%) were single exon circRNAs (Figure 2A). Considering the length of exonic circRNAs, a large number of circRNAs (62.6%) had a size smaller than 500 bp, while a small fraction of circRNAs (1.4%) was over 2000 bp (Figure 2B). These numbers are within the size range previously described for exonic circRNAs (>100 nt to <4 kb) (Lasda and Parker, 2014). In addition, most circRNAs derived from genes that give rise to a single circRNA isoform (77%), as opposed to multiple different isoforms (Figure 2C). The detected circRNAs were present and widely distributed across all rat chromosomes, with exception of the Y chromosome (Figure 2D). This phenomenon has been described by others (Li J. et al., 2018) and might be explained by the low gene density of the Y chromosome across organisms, with the majority of its sequence being composed of non-coding repetitive genome regions that do not produce exonic transcripts (Lluís and Fellous, 2001; Ovcharenko et al., 2005). The majority of detected circRNAs were expressed at low levels at all experimental time points (≤100 normalized read counts) (Figure 2E). Of all circRNAs, 59.4% of the circRNAs were conserved in mouse and had their circRNA ID annotated in the circBase database (Figure 2F). In contrast to previous observations (Rybak-Wolf et al., 2014), only a small portion of the rat circRNAs detected here had a conserved human isoform (16.9%) and a considerable fraction of circRNAs (23.7%) were rat-specific (Figure 2G).

**FIGURE 3:**
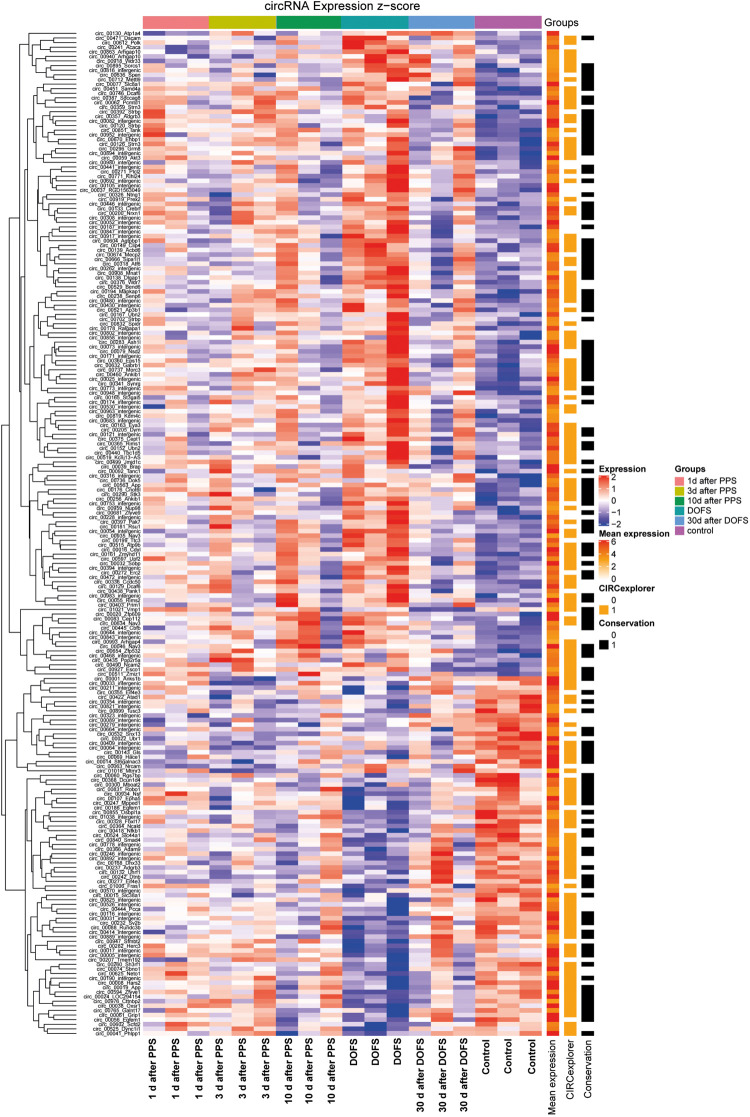
Differentially expressed circRNAs. Heatmap representation of circRNA detection, conservation and expression in the different experimental groups [1 day, 3 days, and 10 days after performant pathway stimulation (PPS); day of first spontaneous seizure (DOFS) and 30 days after DOFS; control]. All circRNAs with FDR < 0.05 for any time point relative to control group are shown. Expression (*z*-score) of each circRNA is represented in each cell, where *z*-score represents the number of standard deviations away from the mean [from –2 (blue) to 2 (red)]. Mean expression shows expression of each individual circRNA across all samples in log scale [low expression (white), medium expression (orange) or high expression (red)]. CIRCexplorer indicates if a specific circRNA is also found by the CIRCexplorer algorithm [found by CIRCexplorer (orange) or not (uncolored)]. Conservation specifies if a circRNA is conserved to mouse, according to circBase [conserved (black) or not conserved (uncolored)].

Out of the 1000 most highly expressed circRNAs, 218 were found to be significantly differentially expressed (DE) across time points (*p* < 0.05) (Figure 3). Most of these 218 circRNAs were also detected by CIRCexplorer, in addition to find_circ (Figure 3). This validation by a second circRNA detection algorithm is important, as annotations for rat genes are currently less well developed as compared to those of for example mouse or human. Only circRNAs detected by both CIRCexplorer and find_circ algorithms were taken into consideration for further experimental analysis.

### Analysis of Differentially Expressed circRNAs at the Day of First Spontaneous Seizure (DOFS)

**FIGURE 4:**
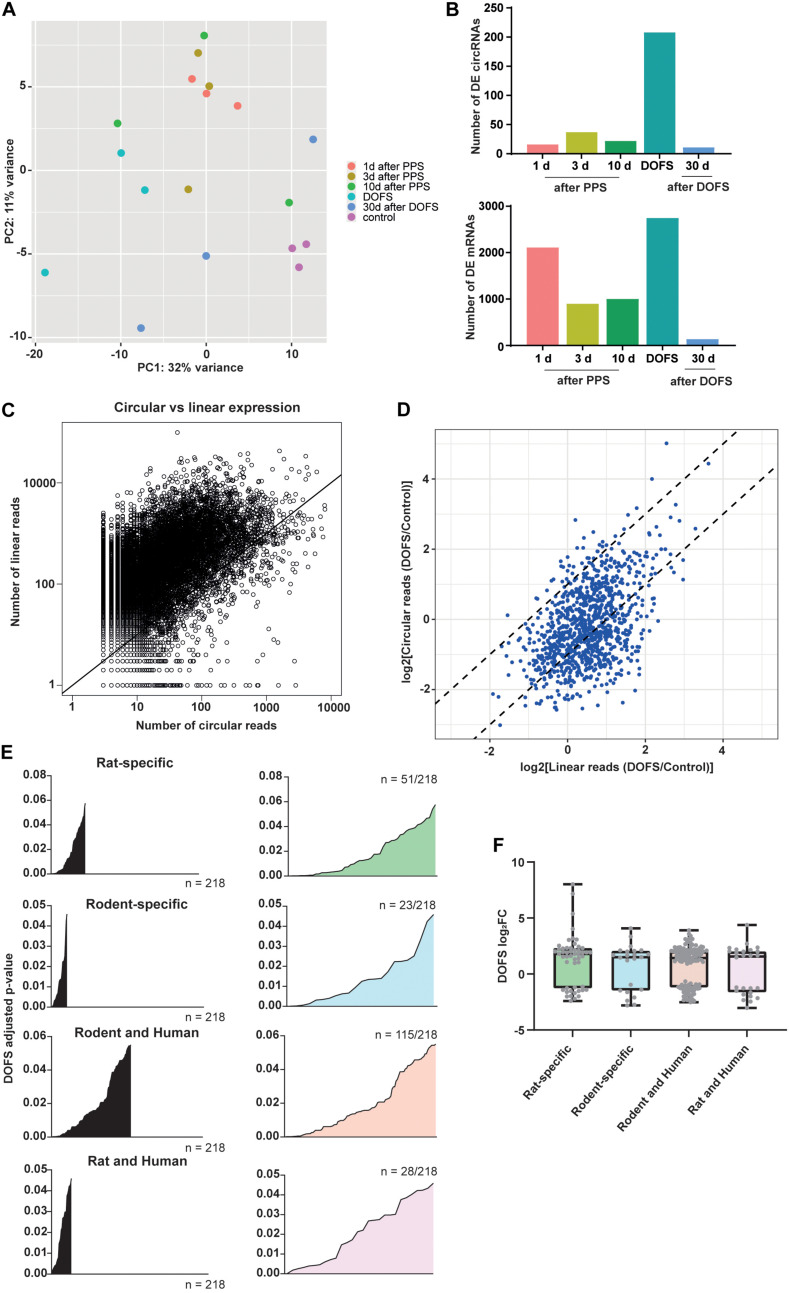
Transcriptome characterization at DOFS. **(A)** Principal component analysis (PCA) of the different experimental groups based on the transcriptome changes of the 1000 most highly expressed circRNAs. **(B)** Number of differentially expressed circRNAs (upper panel) and mRNAs (lower panel) at the six experimental time points (*p* < 0.05) (DE, differentially expressed). **(C)** Scatter plot showing the comparison of circRNA and mRNA expression. The *x*-axis shows the number of reads mapped across the back-splice junction (BSJ) for each detected circRNAs; the *y*-axis shows the number of linearly spliced reads from the corresponding splice acceptor and splice donor sites. Each dot represents one host gene and corresponding circRNA expression. The diagonal black line separates circRNAs that are expressed lower than linear host gene transcripts (above the line) from circRNAs that are expressed higher than linear host gene transcripts (below the line). **(D)** Circular and linear transcript regulation at the DOFS. Axis represent the DOFS/Control ratio for linear (*x*-axis) and circular (*y*-axis) reads. Each dot depicts one circRNA. Dotted lines indicate twofold changes (–2 to 2) and show if differences between control and DOFS impact linear and circular expression equally (in between the two dotted lines) or independently (outside the two dotted lines). **(E)** Statistical significance distribution of the 218 significantly deregulated circRNAs within the three conservation classes (rat-specific = 51/218, rodent-specific = 23/218, rodent and human = 115/218, rat and human = 28/218). **(F)** Distribution of the log_2_FC values of the 218 significantly deregulated circRNAs within the three conservation classes (rat-specific, rodent-specific, rodent and human, rat and human). Each dot depicts one circRNA (log_2_FC, log_2_fold-change).

The circRNA transcriptomes of the six experimental groups were compared using PCA. This showed that although there is overlap between several groups, a clear separation could be observed between the control and DOFS groups (Figure 4A). This observation is supported by the number of DE circRNAs at the different time points. The largest number of DE circRNAs was found at the DOFS, followed by 3 days and 10 days after PPS groups. A similar distribution of DE RNAs was found when analyzing the mRNA transcriptome, which showed the highest number of DE genes at early stages (DOFS and 1 day after PPS) of experimental epilepsy (Figure 4B). Furthermore, our analysis revealed a trend for an overall lower circular-to-linear ratio, i.e., the presence of more mRNA molecules as compared to circRNA molecules per gene (Figure 4C). Considering the observation that major changes in the (non-)coding transcriptome occur at the DOFS, we performed further analyses at this time point. Interestingly, at the DOFS, expression of most circRNAs was linked to host mRNA expression. Nevertheless, a few circRNA changes appeared to be independent of their linear counterparts suggesting that circRNA deregulation is in part uncoupled from host gene expression at the DOFS (Figure 4D). The statistical values of the DE circRNAs at the DOFS were found to be homogeneously distributed over the different categories of species-specific circRNAs (rat-specific, rodent-specific, rodent and human, rat and human) (Figure 4E).

### Differentially Expressed circRNA Selection and Validation

**FIGURE 5:**
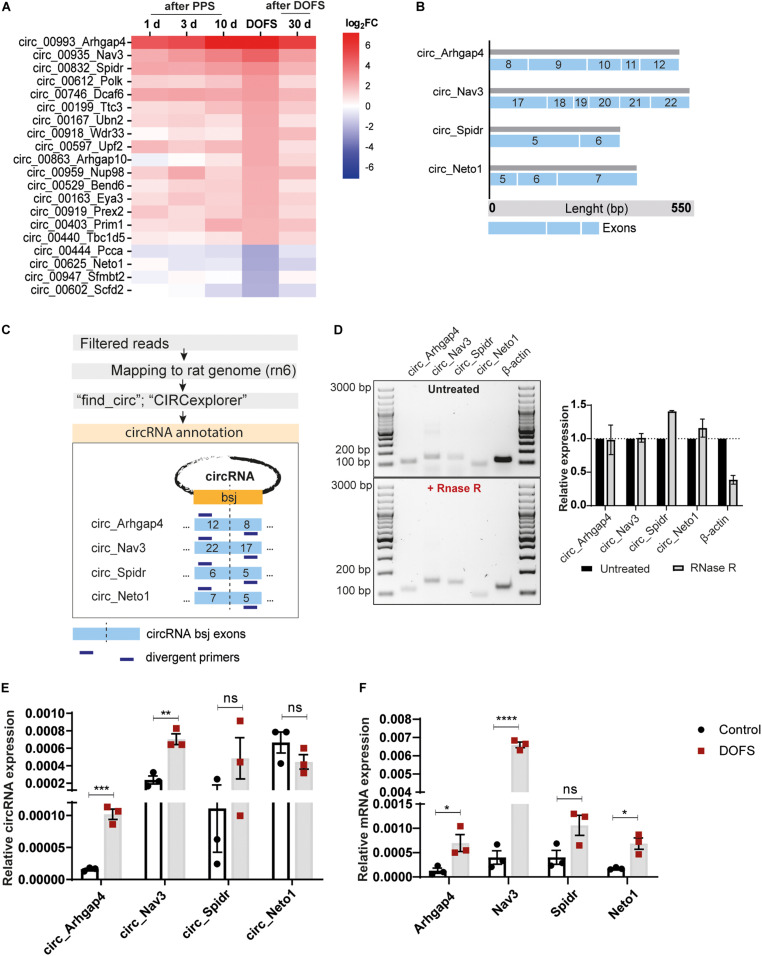
Validation of the expression of selected circRNAs and their linear counterparts at the DOFS. **(A)** Heatmap showing relative expression changes of the most deregulated rat-specific circRNAs. **(B)** Characterization of selected circRNAs (circ_Arhgap4, circ_Nav3, circ_Spidr and circ_Neto1) showing exonic composition, length and exon number. Each blue box represents one exon and the different sizes of the different exonic lengths. **(C)** Schematic workflow of the pipeline used for the annotation of selected circRNAs and subsequent divergent primer design. Two complementary pipelines (“find_circ” and “CIRCexplorer”) were used for the annotation of circRNA sequences. The BSJ of each circRNA was defined as a 100 bp junction between the last and first circRNA exon (blue box). Divergent primers were designed against this region for each circRNA (bsj, back-splice junction). **(D)** Agarose gel electrophoresis of the PCR products from circRNA back-splice junction amplification with (upper panel) and without (lower panel) RNase R treatment. Original images were color inverted for improved visualization. Quantification of RNA expression is shown in the right panel. Values are expressed as the mean ± SD. RNase R-treated expression is normalized to the untreated condition (*n* = 2 independent experiments) (bp, base pairs). **(E)** RT-qPCR analysis of the expression pattern of circRNAs at DOFS (circ_Arhgap4, ***p = 0.0005; circ_Nav3, **p = 0.0038; circ_Spidr, ns; circ_Neto1, ns). The bar plots represent circRNA expression relative to β*-actin* and *Gapdh*. Values are expressed as the mean ± SEM, *p* < 0.001: ***, *p* < 0.01: **, *p* ≥ 0.05: ns (unpaired two-tailed Student’s *t*-test, *n* = 3 animals per group). **(F)** RT-qPCR analysis of the expression pattern of circRNAs’ linear counterparts at DOFS (*Arhgap4*, *p = 0.0354; *Nav3*, ****p < 0.0001; *Spidr*, ns; *Neto1*, *p = 0.0124). The bar plots represent mRNA expression relative to β*-actin* and *Gapdh*. Values are expressed as the mean ± SEM, *p* < 0.0001: ****, *p* < 0.05: *; *p* ≥ 0.05: ns (unpaired two-tailed Student’s *t*-test, *n* = 3 animals per group).

The most prominent deregulation of circRNA expression in the PPS model was observed for rat-specific circRNAs at early stages of epilepsy (DOFS) (Figure 4F). A log_2_ fold-change (log_2_FC) parameter was used as a filtering criterion to select potential circRNA candidates (log_2_FC ≥ 2 or ≤−2). This yielded a total of 20 circRNAs (Table 1) which show a similar pattern of deregulation at the different time points and a marked expression change at the DOFS (Figure 5A). Four candidates (three upregulated and one downregulated) were selected for further validation based on the differential expression between the DOFS and control groups. All of the four selected circRNAs fall into the most common length categories of overall RNA-seq transcripts, with an exonic size below 550 bp. For the selected circRNAs, exon number appeared to be related to the size of the circRNA, with larger circRNAs containing more exons (Figure 5B). Next, we used two circRNA detection bioinformatics tools (“find_circ” and “CIRCexplorer”) to predict the BSJ and further allow circRNA detection (Figure 5C). First, circularity of the four selected circRNAs was assessed by PCR across the BSJ and by RNase R treatment of P1 rat hippocampus RNA. The selected circRNAs were resistant to digestion by RNase R in contrast to a linear reference transcript, β*-actin*, supporting the circular structure of these transcripts (Figure 5D).

**TABLE 1:** List of most differentially expressed circRNAs between control and DOFS groups.

circRNA	DOFS log_2_FC	*P*-value	Genomic region	Host gene description
circ_00993_Arhgap4	7.17	0.0032	chrX:156882943–156884142	Rho GTPase activating protein 4
circ_00935_Nav3	4.38	0.0002	chr7:52223221–52248773	Neuron navigator 3
circ_00832_Spidr	3.18	0.0034	chr11:89175424–89180448	Scaffold protein involved in DNA repair
circ_00612_Polk	2.71	0.0004	chr2:27326040–27333634	DNA polymerase Kappa
circ_00746_Dcaf6	2.70	0.0026	chr13:83601156–83607039	DDB1 and CUL4 Associated Factor 6
circ_00199_Ttc3	2.45	0.0007	chr11:34611538–34614139	Tetratricopeptide repeat domain 3
circ_00167_Ubn2	2.37	0.0040	chr4:66172554–66232564	Ubinuclein 2
circ_00918_Wdr33	2.33	0.0158	chr18:24611732–24614740	WD repeat domain 33
circ_00597_Upf2	2.33	0.0046	chr17:76245626–76265943	Regulator of nonsense transcripts 2
circ_00863_Arhgap10	2.32	0.0125	chr19:34249086–34272145	Rho GTPase activating protein 10
circ_00959_Nup98	2.19	0.0268	chr1:167260357–167269761	Nucleoporin 98 and 96 precursor
circ_00529_Bend6	2.15	0.0042	chr9:38321691–38326937	BEN domain containing 6
circ_00163_Eya3	2.14	0.0005	chr5:150850431–150865550	Eyes absent homolog 3
circ_00919_Prex2	2.08	0.0390	chr5:7703775–7780390	Phosphatidylinositol-3,4,5-trisphosphate dependent Rac exchange factor 2
circ_00403_Prim1	2.03	0.0422	chr7:2440627–2448053	DNA primase subunit 1
circ_00440_Tbc1d5	2.01	0.0402	chr9:1442053–1485363	TBC1 domain family member 5
circ_00444_Pcca	−2.46	0.0018	chr15:109127706–109141568	Propionyl-CoA carboxylase subunit alpha
circ_00625_Neto1	−2.41	0.0072	chr18:83542805–83556790	Neuropilin and tolloid like 1
circ_00947_Sfmbt2	−2.21	0.0377	chr17:71875847–71886141	Scm like with four Mbt domains 2
circ_00602_Scfd2	−2.15	0.0147	chr14:36341463–36341713	Sec1 family domain containing 2

Next, the relative expression levels of the four selected circRNAs were validated in control and DOFS rat samples using qRT-PCR and divergent primer sets. This qRT-PCR analysis was generally in line with the RNA-seq data. Specifically, circ_Arhgap4 and circ_Nav3 were found to be significantly upregulated in the DOFS group (circ_Arhgap4, ^∗∗∗^*p* = 0.0005; circ_Nav3, ^∗∗^*p* = 0.0038) as compared to control (Figure 5E). Although circ_Spidr and circ_Neto1 also appeared to be deregulated, these changes were not statistically significant (Figure 5E). The linear host transcripts for circ_Arhgap4, circ_Nav3 and circ_Neto1 were significantly increased (*Arhgap4*, ^∗^*p* = 0.0354; *Nav3*, ^****^p < 0.0001, *Neto1*, ^∗^*p* = 0.0124) at DOFS (Figure 5F).

### circRNA/miRNA/mRNA Networks

**FIGURE 6:**
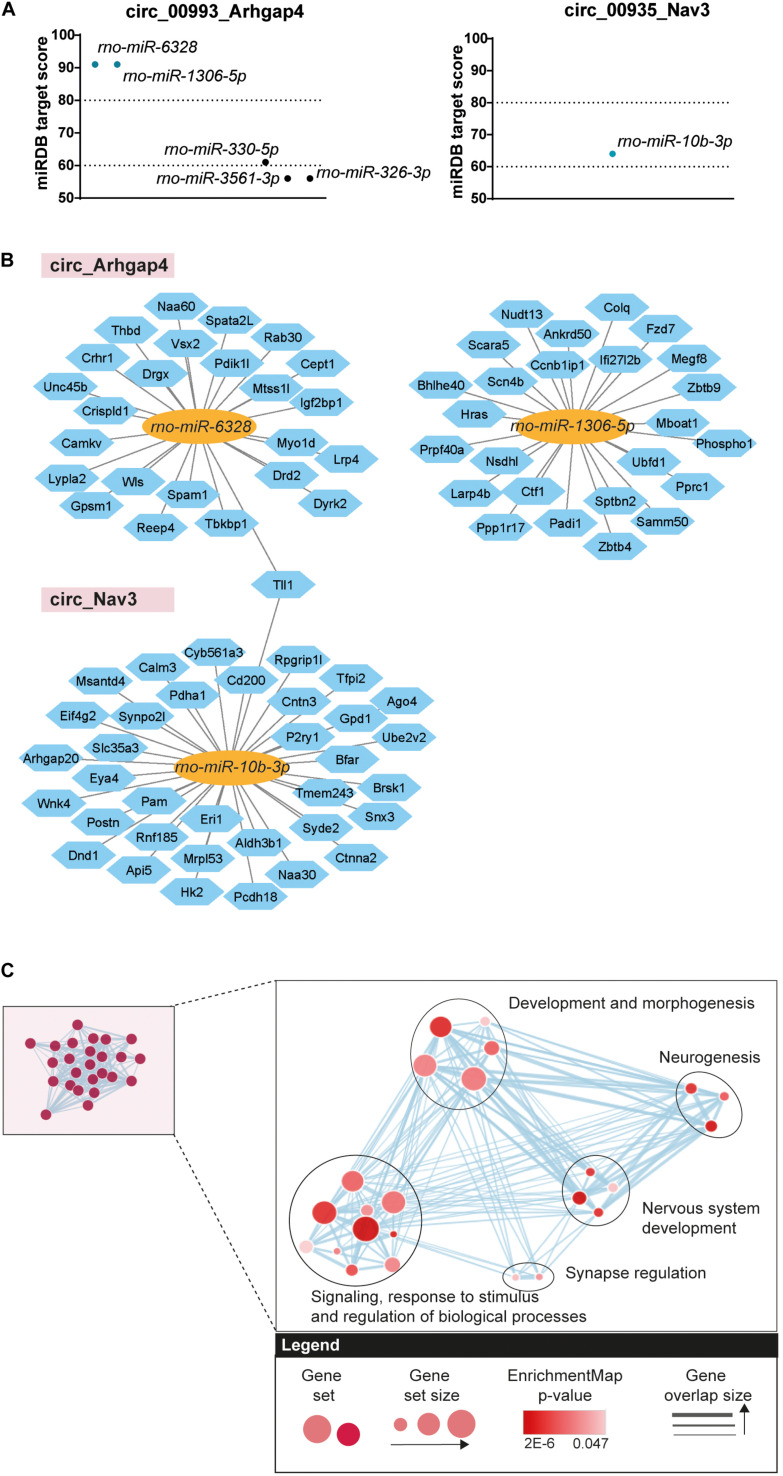
Bioinformatics analysis of circRNA/miRNA networks. **(A)** Predicted miRNA targets of circ_Arhgap4 and circ_Nav3 (miRDB predicted score > 60). Dotted lines represent high confidence (miRDB predicted score = 80) and minimum confidence (miRDB predicted score = 60) thresholds used in further selection. **(B)** Diagram showing predicted mRNA targets derived from the circRNA/miRNA (miRDB analysis, predicted score > 80). circRNAs are represented by the pink rectangles; predicted bound miRNAs by the yellow ovals and miRNA targets by the blue hexagons. **(C)** Cluster representation of GO biological processes associated with the selected circRNA/miRNA interactions. Different GO categories were assigned into clusters according to their biological function. Each black circle represents a cluster; the colored circles represent groups of genes enriched in a certain GO category; the size of the circle denotes the number of genes composing that GO category; the color gradient shows significance and the gray arrows represent gene overlap between categories (FDR < 0.05).

As upregulation of circ_Arhgap4 and circ_Nav3 at the DOFS was confirmed by qRT-PCR we further focused on these circRNAs. We performed custom DNA sequencing of circ_Arhgap4 and circ_Nav3 BSJs as an extra validation of their sequences, which confirmed the RNA-seq annotations (Supplementary Figures 1A,B). Next, we examined possible downstream effects of circ_Arhgap4 and circ_Nav3 upregulation at the DOFS. Accumulating evidence reports a role for circRNAs in regulating gene expression via specific circRNA/miRNA/mRNA-associated networks (Rong et al., 2017; Kristensen et al., 2019; Guria et al., 2020). This results from the ability of circRNAs to compete with mRNAs for miRNA binding via miRNA response elements (MREs) (Hansen et al., 2013; Memczak et al., 2013; Thomas and Sætrom, 2014). Therefore, miRDB was used to predict miRNA binding sites in the selected circRNAs. Given the circular nature of circRNA transcripts and their rat-specificity, the custom prediction tool from miRDB was used to obtain the predicted sequences of miRNA interactions with the circRNA spliced sequence, including the back-splice region. Circ_Arhgap4 and circ_Nav3 harbored five or one, respectively, predicted miRNA sites and one to three binding sites for each miRNA (Supplementary Table 4). A miRDB prediction score > 60 was considered as an inclusion criterion and following application of this cutoff one and two miRNAs were selected for circ_Arhgap4 (*rno-miR-6328* and *rno-miR-1306b-5p*) and circ_Nav3 (*rno-miR-10b-3p*) (Figure 6A). We further performed target prediction analysis to identify potential targets of the selected miRNAs. After applying a prediction score threshold ≥ 80, an average of 25 mRNA targets were found for each miRNA (Figure 6B) (listed in Supplementary Table 4). One mRNA target, *tolloid-like-1* (*Tll1*) was shared between the potential miRNA binding partners of circ_Nav3 and circ_Arhagp4. Next, we probed the involvement of predicted circRNA/miRNA targets in key biological processes. g:Profiler was used to perform functional enrichment of the mRNA targets. Retrieved GO categories were grouped based on a higher level of clustering and graphically represented using EnrichmentMap Cytoscape App (Figure 6C). Full annotations and subsequent clustering are listed in detail in Supplementary Table 5. Signaling, response to stimulus and regulation of biological processes, development and morphogenesis, neurogenesis, nervous system development and synapse regulation were found to be among the most significant pathways associated with the circRNA/miRNA targets (Figure 6C).

**FIGURE 7:**
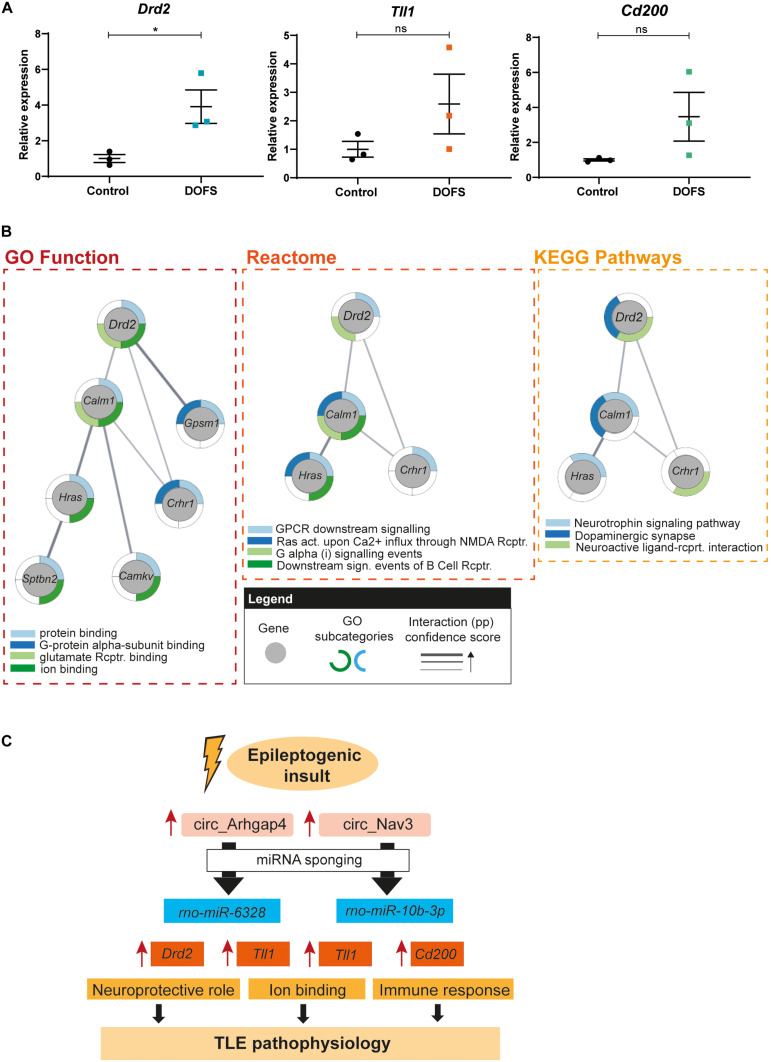
*Drd2* network pathway analysis and schematic model of proposed circRNA/miRNA/mRNA networks. **(A)** RT-qPCR analysis of the expression of selected mRNAs at day of first spontaneous seizure (DOFS). *Drd2* is significantly upregulated at DOFS (*p = 0.0395). Data are expressed as the mean ± SEM normalized to the control condition, *p* < 0.05: *; *p* ≥ 0.05: ns (unpaired two-tailed Student’s *t*-test, *n* = 3 animals per group). **(B)** GO function, reactome and KEGG analysis of the *Drd2* interactome. Gray circles show each of the genes composing the network; outside colored layers illustrate the different GO subcategories associated with each gene and gray lines represent the confidence score related to the protein–protein interactions (pp) of the targets composing the network (act., activation; rcptr., receptor). **(C)** Proposed model of the circRNA/miRNA/mRNA pathways explored in this study. Increase of circ_Arhgap4/circ_Nav3 expression in response to an epileptogenic insult may regulate the expression of miRNA targets via sponging. A consequent reduction in miRNA availability leads to an increase in miRNA targeted transcripts with specific molecular functions. Neuroprotection (*Drd2*), ion binding (*Tll1*) and immune response (*Cd200*) are a few of the functions through which these deregulated transcripts may contribute to TLE pathophysiology following PPS. Blue rectangles display miRNAs predicted to bind the selected circRNAs; orange rectangles show predicted miRNA targets and yellow rectangles represent molecular mechanisms known to be regulated by the miRNA target genes.

circRNA/miRNA interactions have been proposed to control miRNA availability and therefore indirectly control the expression of downstream miRNA targets (Panda, 2018). Enhanced expression of a circRNA may therefore result in decreased availability of miRNAs and thereby increased expression of mRNAs targeted by these miRNAs. To test this hypothesis for the circRNAs studied here, the expression of three miRNA targets was assessed by qRT-PCR in control and DOFS samples. *Tll1*, *dopamine receptor D2* (*Drd2*) and *cluster of differentiation 200* (*Cd200*) were selected because of their reported link to epilepsy, shared interactions and molecular functions (Table 2). Consistent with the proposed model, expression of the three mRNA targets was increased at the DOFS, although this effect was only statistically significant for *Drd2* as compared to control (Figure 7A). *Drd2* is one of the top circ_Arhgap4:*miR-6328* target genes (target score = 83). Further, it was found to be enriched in all of the 24 GO terms retrieved for the miRNA target gene set (Supplementary Table 5). StringApp was used to functionally characterize and visually represent *Drd2* in three categories: GO Molecular Function, Reactome and KEGG Pathways. This approach allowed an integrative perspective of *Drd2* functions while considering *Drd2* protein interactions with the remaining miRNA targets in the network (pp). All results from the *Drd2* network analysis are listed in Supplementary Table 6. The most representative categories linked to *Drd2* included protein binding, binding to glutamate receptor, ion binding, GPCR downstream signaling, G alpha (i) signaling events, neuroactive ligand-receptor interaction, and dopaminergic synapse (Figure 7B). Moreover, *Drd2* can act as an effector with additional functions in different pathways (G-protein alpha-subunit binding, Ras activation upon Ca^2+^ influx through NMDA receptor, downstream signaling events of B Cell receptor and neurotrophin signaling pathway) via pp interactions with other network targets (Figure 7B). Overall, the functional characteristics of the *Drd2* network are mainly associated with the intracellular transduction of extracellular signals (ion binding, GPCR activation or neurotrophin signaling), key players in processes like memory, learning and synaptic plasticity (Reichardt, 2006; Voglis and Tavernarakis, 2006; Calebiro et al., 2010). A closer inspection of the retrieved GO categories revealed both glutamate and dopamine signaling. Glutamate receptors are the main excitatory mediators in the brain and their overactivation has been associated with epileptogenesis (Barker-Haliski and Steve White, 2015). Additionally, dopaminergic receptors are known to play a role in the modulation of seizures that originate in the limbic system (Bozzi and Borrelli, 2013). The above described bioinformatics results suggest an important role for *Drd2* in initiating a physiological response in the brain and, particularly, during epileptogenesis.

**TABLE 2:** List of circRNA/miRNA/mRNA targets with a reported role in epilepsy.

Target	Gene description	Role in epilepsy	References
*Drd2*	Dopamine receptor D2	Increased hippocampal death in *Drd2*^–/–^ mice	Bozzi et al. (2000)
*Tll1*	Tolloid-like 1	Increased transcript degradation by NMD upon SE	Mooney et al. (2017)
*Cd200*	Cluster of differentiation 200	mRNA downregulation in epileptogenic lesions of Focal cortical dysplasia type IIb	Sun et al. (2016)

## Discussion

The search for novel and effective therapeutic targets for the treatment of epilepsy is a priority in the field. In the past years, increasing experimental evidence has identified ncRNAs as potential therapeutic targets in epilepsy (Henshall and Kobow, 2015; Shao and Chen, 2017; Mills et al., 2020). Different types of ncRNAs exist and here we focused on circRNAs, a recently rediscovered class of ncRNAs, with enriched expression in the brain and the ability to regulate gene expression via different mechanisms. Thus far, studies on the role of circRNAs in epilepsy have focused on chronic stages of TLE, both in patient samples and in a mouse model (Gong et al., 2018; Lee et al., 2018; Li J. et al., 2018). Here, we used RNA-seq to explore circRNA expression at different stages from the initiation of experimental TLE through to the chronic stages, when spontaneous seizures are well established. Interestingly, deregulation of circRNA expression was most prominent at the DOFS, with 218 DE circRNAs. Another recent study using a mouse model of epilepsy detected 48 DE genes at 60 days after pilocarpine-induced SE (chronic epilepsy) (Lee et al., 2018). Although this study used microarray technology to probe circRNA expression, the limited number of differently expressed circRNAs at late (and very early) stages of experimental epilepsy in mouse and rat models may hint at specific regulation of circRNA expression during the transition from epileptogenesis to chronic epilepsy. In our study, the majority of deregulated circRNAs (68%) displayed increased expression. This is consistent with observations from a recent study using a pilocarpine mouse model (60.5% upregulated circRNAs) (Lee et al., 2018). In contrast, in brain tissue from TLE patients, circRNA expression was predominantly downregulated among the DE targets (57.5% downregulated circRNAs) (Li J. et al., 2018). This apparent discrepancy may be explained by the fact that brain material from TLE patients often represents the “end stage” of the disease, which is characterized by massive cell death and other cellular changes that could cause reduced tissue expression of specific circRNAs and which typically occurs after several decades of uncontrolled epilepsy.

Multiple studies have reported circRNA expression regulation independent of that of the corresponding linear transcripts (Jeck et al., 2013; Ashwal-Fluss et al., 2014; Rybak-Wolf et al., 2014; Xu et al., 2018). However, also concomitant changes in the expression of both types of RNA are reported, e.g., during neuronal differentiation (Salzman et al., 2013; Rybak-Wolf et al., 2014). We validated the upregulation of circ_Arhgap4 and circ_Nav3 at the DOFS and found similar regulation of their circular and linear host transcripts. This is in contrast with earlier findings, in which no obvious correlation was observed between specific circRNAs and the expression of their parental genes in mTLE (Gray et al., 2020). A positive correlation in the expression of circRNA and mRNA species suggests that the RNA changes are a consequence of transcriptional activation of these genes rather than an alteration of splicing events that lead to circRNA formation (Jeck and Sharpless, 2014). Further, it may indicate a role for these specific circRNAs in regulating host gene expression (Barrett and Salzman, 2016). Since we were able to replicate the predicted RNA-seq changes of circ_Arhgap4 and circ_Nav3 by RT-qPCR analysis we decided to focus on these two candidates. Although we focused on rat-specific and rat and human conserved circRNAs, given the fact that those were the most deregulated circular transcripts, circ_Nav3 isoforms other than the one studied were detected by RNA-seq and found to be differentially expressed in DOFS samples. These isoforms are conserved in rodents and humans, which highlights the relevance of *Nav3* circular transcripts in epilepsy and could be further explored.

Mossy fiber growth of DG granule cells and reactive astrogliosis are hallmarks of TLE. Aberrant sprouting is characterized by the projection of mossy fibers from the hilus through the granule layer thereby re-innervating other regions of the DG (Proper et al., 2000). This feature can be found in TLE patients but also in rats that are treated with kainic acid or pilocarpine, and in the PPS rat model used in our study (Tauck and Nadlers, 1985; Okazaki et al., 1999; Proper et al., 2000; Norwood et al., 2010). This mossy fiber sprouting enhances the excitatory loop in dentate granule cells facilitating electrical discharges and, eventually, seizure propagation (Okazaki et al., 1999). Additional observations from tissue of epileptic patients and animal models of epilepsy show that glial cells undergo marked morphological and biochemical alterations that are associated with changes in the expression of astrocytic ion channels (Niquet et al., 1994; Beach et al., 1995; Jacobs et al., 1996; Bordey and Sontheimer, 1998). Interestingly, *Arhgap4* encodes for ARHGAP4, a Rho GTPase activating protein (GAP) that inhibits hippocampal axon growth following overexpression (Vogt et al., 2007). NAV3 is expressed in the outer nuclear membrane of neurons and its expression is induced in reactive astrocytes and neurons following brain injury (Coy et al., 2002). It is therefore possible that the observed enhanced expression of *Arhgap4* and *Nav3* transcripts may contribute to the pathogenesis of TLE. While additional experimental work is needed to test this model, we further explored a possible role of enhanced circ_Arhgap4 and circ_Nav3 expression using gene expression analysis and bioinformatics approaches.

Several circRNAs have been shown to act as miRNA sponges via MRE sites (Hansen et al., 2013; Memczak et al., 2013; Thomas and Sætrom, 2014). Our analysis showed that circ_Arhgap4 and circ_Nav3 are predicted to interact with *miR-6328* and *miR-10b-3p*, respectively, and may therefore indirectly regulate expression of *miR-6328* and *miR-10b-3p* targets. Notably, neither *miR-6328* nor *miR-10b-3p* were found among the DE miRNAs at DOFS (data not shown), suggesting sponging by circ_Arhgap4 and circ_Nav3 does not impact miRNA expression in this specific context. *miR-6328* was identified as a rat-specific miRNA derived from intronic regions of chromosome 20 and its functions are unknown (Clokie et al., 2012). On the other hand, the expression of *miR-10b-3p*, conserved across rodents and humans, is increased in rat brains undergoing stress-induced depression or decreased in rats exposed to cold stress and in regions of brain damage following ischemia (Zhen et al., 2017; Duan et al., 2019; Fang et al., 2020). Even though no link between *miR-10b-3p* and epilepsy has been established so far, the *5p* strand of *miR-10b* is linked to epilepsy. *miR-10b-5p* expression is deregulated in several epilepsy animal models and patients (Liu et al., 2010; Hu et al., 2011; Jimenez-Mateos et al., 2012; Kaalund et al., 2014), suggesting a function for the *miR-10* gene family in TLE.

To investigate a potential involvement of circ_Arhgap4 and circ_Nav3, and their predicted miRNA targets in TLE, bioinformatics analysis was used to identify potential *miR-6328* and *miR-10b-3p* targets followed by network analysis of the circRNA/miRNA/mRNA axis. The GO terms retrieved by the functional enrichment analysis of *miR-6328/miR-10b-3p* targets included ‘signaling,’ ‘response to stimulus,’ and ‘regulation of biological processes.’ A possible explanation for this finding is that the observed changes reflect a response to circRNA deregulation as a result of altered neuronal activity. This is in line with the observed enhanced expression of circRNAs at the DOFS and with several studies reporting the regulation of circRNA expression in response to neuronal activity and their synaptic enrichment (Rybak-Wolf et al., 2014; You et al., 2015; Xu et al., 2018). Of the predicted miRNA targets, we found significant upregulation of *Drd2* at the DOFS. *Drd2* encodes dopamine receptor D2 (D2R) and its expression is predicted to be controlled by *miR-6328*, a miRNA bound by circ_Arhgap4 (Bunzow et al., 1988; Dal Toso et al., 1989). GO Function, Reactome and KEGG pathway analyses revealed an association of *Drd2* with glutamate receptor binding, signaling events, dopaminergic synapse and neuroactive ligand-receptor interactions. Regulation of D2R surface expression in the DG has been linked to synaptic plasticity and memory formation in mice, processes known to be deregulated in epilepsy (Reid and Stewart, 1997; Saab et al., 2009). The specific role of D2 receptors in epilepsy has been extensively investigated in both animal and human studies (Starr, 1996). Inhibition of glutamatergic neurotransmission, susceptibility to KA-induced seizures and regulation of excitotoxicity in the brain are some of the mechanisms previously associated with D2R deregulation. Previous reports have shown that the absence of D2R (*Drd2*^–^*^/^*^–^ mice) leads to a lower seizure threshold and increased neurotoxicity in response to treatment with both kainic acid and pilocarpine in comparison to wild-type mice (Bozzi et al., 2000; Bozzi and Borrelli, 2002). These data support the idea that D2R may act as a neuroprotective agent by reducing seizure activity and extensive hippocampal neuronal death (Starr, 1996; Bozzi et al., 2000; Bozzi and Borrelli, 2002; Brodie et al., 2016). Increased expression of *Drd2* as reported here may reflect a protective response against PPS-induced neurodegeneration during early epileptogenesis. Overall, our data supports a model in which upregulation of circ_Arhgap4 may reduce the availability of *miR-6328* leading to an increase of D2R, which may trigger protection against epilepsy-associated changes.

Most circRNAs identified here only contain one or a few predicted miRNA binding sites. With the exception of Cdr1as (which contains over 70 *miR-7* binding sites) only few circRNAs contain multiple binding sites for a particular miRNA (Guo et al., 2014). Currently, it is suggested that the abundance of circRNA and miRNA should be similar in order for the sponging process to be considered effective (Guria et al., 2020). This mechanism can be achieved either by a high number of binding sites for a specific miRNA or by an increase in the number of circRNA copies (Zheng et al., 2016). Interestingly, subsets of circRNAs can be highly enriched in cellular compartments, such as synapses (Rybak-Wolf et al., 2014; You et al., 2015), thereby creating an increased local concentration. It is also possible that the circ_Arhgap4/circ_Nav3 axis exerts an effect on *Drd2* and other targets via different mechanisms than miRNA sponging, such as binding to RBPs or transcriptional regulation (Lasda and Parker, 2014). As analysis of regulatory circRNA/miRNA/mRNA networks relies on bioinformatics and predictions further work is necessary to explore the effects of circ_Arhgap4 on *miR-6328* expression levels and unravel the biological processes associated with the circ_Arhgap4/*miR-6328*/*Drd2* network.

## Conclusion

Our study is the first to profile circRNA expression at different stages of experimental epilepsy. We show that circRNA expression is actively regulated during the process of epileptogenesis leading to epilepsy. These observations support a model in which enhanced neuronal activity due to the occurrence of seizures triggers the expression of circRNAs which could ultimately contribute to the pathogenic process of TLE (Figure 7C). The events surrounding the transition of a post-insult but pre-epileptic network to a brain thereafter generating recurrent spontaneous seizures are poorly understood. Our results indicate that circRNA expression is markedly deregulated during this transition. Interestingly, circRNAs and mRNAs but not miRNAs show such enrichment (Venø et al., 2020). If changes in circRNA expression are an essential feature of the transition period it offers the potential to intervene at this stage and prevent progression to epilepsy.

## Data Availability Statement

The datasets generated for this study are deposited in the Gene Expression Omnibus (GEO) under the accession number GSE137473.

## Ethics Statement

The animal studies were reviewed and approved by Animal Ethics Committee of Philips University Marburg, Germany or by Utrecht University, Netherlands.

## Author Contributions

AG-D contributed to the methodology, investigation, formal analysis, visualization, and writing – original draft preparation. SB contributed to the methodology, conceptualization, animal work, investigation, and writing – review and editing. BN contributed to the methodology, and writing – review and editing. MV contributed to the analysis and visualization. DCH, JK, and FR contributed to the resources and writing – review and editing. VRV contributed to the methodology, investigation, formal analysis, visualization, and writing – review and editing. RJP contributed to the conceptualization, resources, writing – review and editing, supervision, and funding acquisition. All authors contributed to the article and approved the submitted version.

## Conflict of Interest

MV was employed by the company Omiics ApS. BN was employed by the companies Expesicor Inc. and FYR Diagnostics. The remaining authors declare that the research was conducted in the absence of any commercial or financial relationships that could be construed as a potential conflict of interest.

## Footnotes

1 http://circbase.org/

2 https://www.ncbi.nlm.nih.gov/tools/primer-blast/

3 http://www.mirdb.org

4 http://www.mirdb.org/custom.html

5 https://biit.cs.ut.ee/gprofiler/gost

## Supplementary Material

Supplementary Figure 1 Sequencing validation of circ_Arhgap4 and circ_Nav3 back splice junctions. Custom DNA sequencing following RT-qPCR was used to confirm the sequence identity of the back splice junctions of **(A)** circ_Arhgap4 and **(B)** circ_Nav3 (BSJ, back splice junction).

Supplementary Table 1 circRNAs found after DESeq2 analysis. All circRNAs detected after RNA-seq and found to be differentially expressed by DESeq2 analysis are listed. circRNAs are sorted based on the adjusted *p*-values at DOFS. *N* = 3 animals were used in each group (MRP2, 1 day after PPS; MRP3, 3 days after PPS; MRP4, 10 days after PPS; MRP6, DOFS; MRP7, 30 days after DOFS; MRP8, control; padj, adjusted *p*-value).

Supplementary Table 2 List of oligonucleotides and corresponding sequences used in this study. Primers used for RT-qPCR were designed using the Primer-BLAST online tool (https://www.ncbi.nlm.nih.gov/tools/primer-blast/) or were obtained from previous publications (*Spidr, Cd200*) (Fonken et al., 2018; Mao et al., 2019). The following criteria were applied during the design: oligonucleotide length 18–24 bp; melting temperature (Tm) 52–64°C; %G/C content 40–60%; minimum self-complementary regions and generation of an amplicon size < 250 bp. All primers were purchased from IDT-DNA.

Supplementary Table 3 NormFinder analysis to identify normalization genes. NormFinder (^∗^.xla, MS Excel 2003 v0.953) statistics was used determine the most stable reference gene combination considering the two experimental groups (control and DOFS). NormFinder analysis focused on the intra- and inter-individual expression variation of the transcripts. The best combination of genes is provided given the calculated stability value: the lower the value, the more stable the combination (β*-actin* and *Gapdh* = 0,188). Raw untransformed cycle threshold (Ct) values were used and group identifiers (group id) given to the different groups (C1–3, control = 0, DOFS1–3, DOFS = 1). A spike in derived from *Caenorhabditis elegans* (c.e.) was used during cDNA synthesis in order to control for possible artifacts.

Supplementary Table 4 MiRDB analysis of predicted miRNA binding sites in the circRNA sequence. MiRDB database was used to perform bioinformatics analysis of circRNA and miRNA targets. Initially, ‘miRNA custom prediction tool’ was used to predict miRNA binding sites (or MREs) present in the circRNA sequences, back-splice junction included. Only predicted miRDB scores > 60 were considered. Selected *rno-miRNAs* were then individually used in the ‘target search tool.’ Several miRNA targets were retrieved for each miRNA, with predicted 3′UTR binding sites. Only predicted miRDB scores ≥ 80 were considered.

Supplementary Table 5 g:Profiler analysis of circRNA/miRNA/mRNA targets. g:GOSt tool of g:Profiler was used to perform functional profiling of the circRNA/miRNA/mRNA targets retrieved by miRDB (miRDB score ≥ 80). Cytoscape interface (Enrichment map) was used to identify GO categories over-represented among the targets. ‘Node table > Enrichment’ represents all targets found to be enriched in a certain GO class and correspondent statistics. ‘Edge table > gene overlap’ contains all genes found to be overlapping targets among the GO categories. Existent GO categories were classifying and grouped into five general classes according to their functional profiling (“nervous system development,” “development and morphogenesis,” “neurogenesis,” “synapse regulation,” and “signaling, response to stimulus and regulation of biological processes”) and graphically visualized in Cytoscape. Enrichment was calculated as −log_10_fdr_qvalue and enrichment significance considered for fdr_qvalue < 0.05 (Node table: EnrichmentMap GS_DESCR, gene set description; EnrichmentMap:GS_Type, enrichment nodes; EnrichmentMap GS size, number of genes found in each gene set/GO category; fdr_qvalue, gene set q-value. Edge table: shared name, overlapping gene sets/GO categories; EnrichmentMap:similarity_coefficient, calculated coefficient of the overlap between the two gene sets/GO categories; EnrichmentMap:Overlap_size, number of genes associated with the overlap of the GO categories).

Supplementary Table 6 Complete annotations of *Drd2* network analysis (KEGG, reactome, GO function). Cytoscape StringApp was used to perform functional enrichment analysis of *Drd2* and interacting genes. The entry list included all circRNA/miRNA/mRNA targets retrieved by miRDB (miRDB score ≥ 80) but only the networks including *Drd2* were considered. All retrieved categories (reactome, KEGG pathways and GO function) subcategories’ description and genes found are listed. Enrichment was calculated as log_10_FDR and statistical significance considered for FDR < 0.05 (GO, gene ontology; FDR, false discovery rate).

## Abbreviations

act-1: actin-1 precursor
AEDs: anti-epileptic drugs
BSJ: back-splice junction
CA1: *Cornu Ammonis* 1
CA3: *Cornu Ammonis* 3
*Cd200*: cluster of differentiation 200
cDNA: complementary DNA
ceRNAs: competitive endogenous RNAs
circRNA: circular RNA
Ct: cycle threshold
c.e.: *C. elegans*
DE: differentially expressed
DG: dentate gyrus
dH2O: distilled water
DOFS: day of first spontaneous seizure
*Drd2*: dopamine receptor D2
FC: fold-change
FDR: false discovery rate
*Gapdh*: glyceraldehyde-3-phosphate dehydrogenase
GO: gene ontology
HS: hippocampal sclerosis
ILAE: International League Against Epilepsy
i.p.: intraperitoneal
KEGG: Kyoto Encyclopedia of Genes and Genomes
log_2_FC: log_2_fold-change
miRNA: microRNA
MREs: microRNA response elements
mRNA: messenger RNA
mTLE: mesial temporal lobe epilepsy
ncRNAs: non-coding RNAs
NMD: nonsense mediated decay
padj: adjusted *p*-value
PCA: principal component analysis
PCR: polymerase chain reaction
PP: perforant pathway
PPS: perforant pathway stimulation
RBPs: RNA binding proteins
RISC: RNA-induced silencing complex
rn6: rat genome
RNA-seq: RNA-sequencing
Rnase R: ribonuclease R
rRNA: ribosomal RNA
RT-PCR: reverse transcriptase PCR
RT-qPCR: reverse transcriptase quantitative PCR
SD: standard deviation
SE: status epilepticus
SEM: standard error of the mean
TGF: transforming growth factor
TLE: temporal lobe epilepsy
*Tll1*: tolloid-like-1
UTRs: untranslated region
β *-actin*: beta-actin.
