# Supplementary figures and images for: Enrichment of Circular RNA Expression Deregulation at the Transition to Recurrent Spontaneous Seizures in Experimental Temporal Lobe Epilepsy
Source: Front Genet. 2021 Jan 28;12:627907. doi: 10.3389/fgene.2021.627907 (PMC7876452; doi:10.3389/fgene.2021.627907)

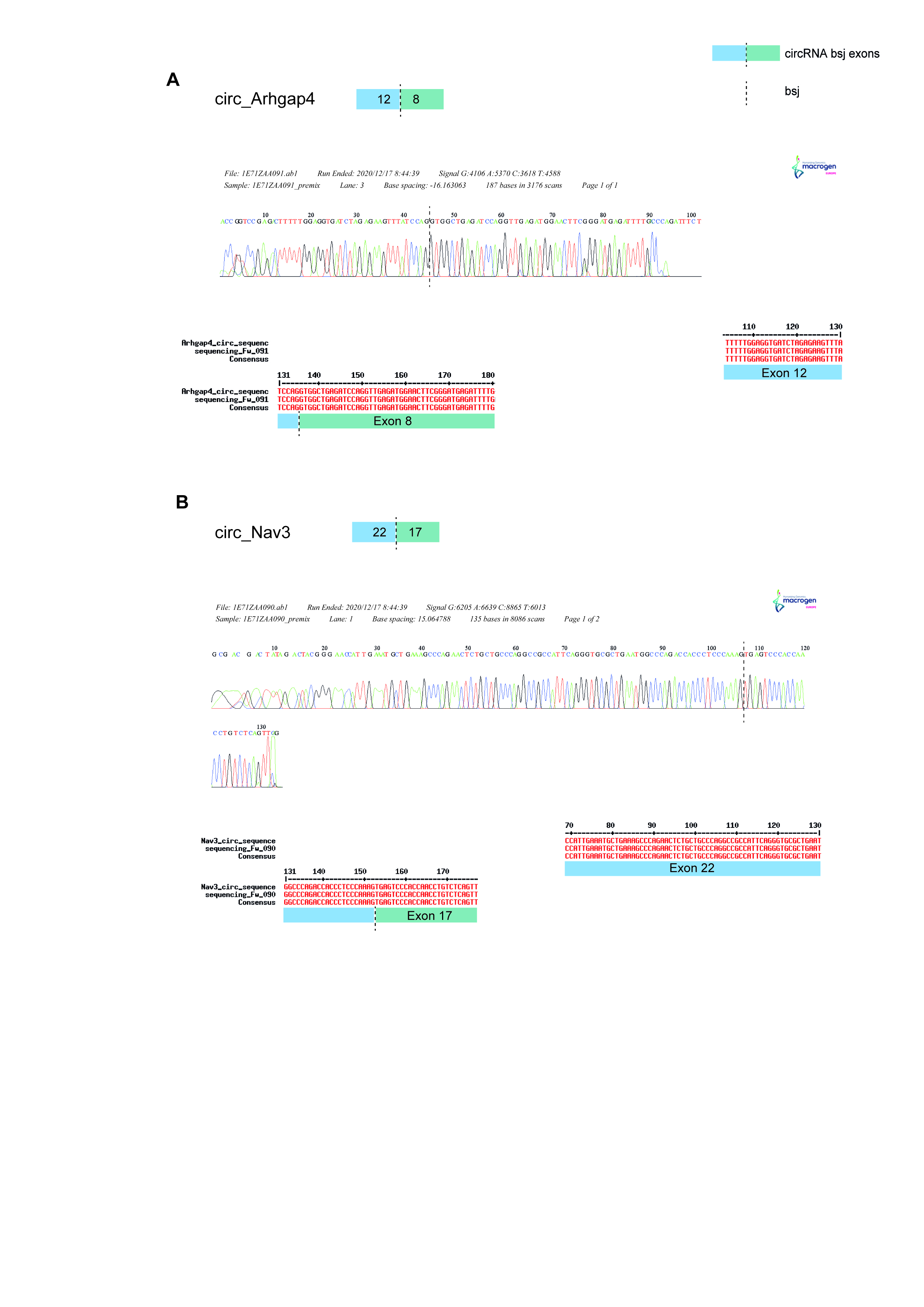

Supplement: Supplementary Figure 1 — Sequencing validation of circ_Arhgap4 and circ_Nav3 back splice junctions. Custom DNA sequencing following RT-qPCR was used to confirm the sequence identity of the back splice junctions of (A) circ_Arhgap4 and (B) circ_Nav3 (BSJ, back splice junction). [file Image_1.jpg]
